# Availability of Radiopharmaceuticals and Imaging Equipment in English-Speaking African Countries

**DOI:** 10.2967/jnmt.125.270432

**Published:** 2025-12

**Authors:** Lerato S. Mosima, Amanda E. Manicum, Beverley Summers

**Affiliations:** 1 Department of Pharmaceutical Sciences, Sefako Makgatho Health Sciences University, Gauteng, South Africa; and; 2 Department of Chemistry, Tshwane University of Technology, Gauteng, South Africa

**Keywords:** radiopharmaceuticals, English-speaking Africa, noncommunicable disease, SPECT, PET

## Abstract

Radiopharmaceutical services are key in cancer screening, diagnosis, staging, treatment monitoring, detection of remission, and therapy. Unfortunately, due to the high costs of these services, their availability is very limited in developing countries. This study highlights issues related to access to radiopharmaceuticals and imaging equipment in English-speaking African countries. **Methods:** The study used a cross-sectional, quantitative online survey approach. **Results:** Findings revealed that only 13 of the 24 English-speaking African countries have nuclear medicine sites; 62% of countries with nuclear medicine sites rely on sole suppliers for ^99^Mo/^99m^Tc generators. Of these countries, South Africa, Tanzania, Uganda, Zambia, and Zimbabwe procure their generators only from South Africa, whereas Kenya, Mauritius, and Sudan procure theirs solely from European countries and Turkey. Cameroon, Ethiopia, Ghana, Namibia, and Nigeria procure generators from multiple countries. South Africa is the only English-speaking African country that commercializes radiopharmaceuticals and therefore has the greatest access to radiopharmaceuticals in this region. Only 23% of English-speaking African countries having access to PET services and theranostics. **Conclusion:** Gaps in access to radiopharmaceuticals among English-speaking African countries have been identified and are largely due to the lack of equipment and poor infrastructure. Plans to strengthen access to radiopharmaceuticals in some of the countries in this region are under way. Our survey found that 331,758,822 people from 11 English-speaking African countries from the region have no access to radiopharmaceutical services.

## Visual Abstract

**Figure:**
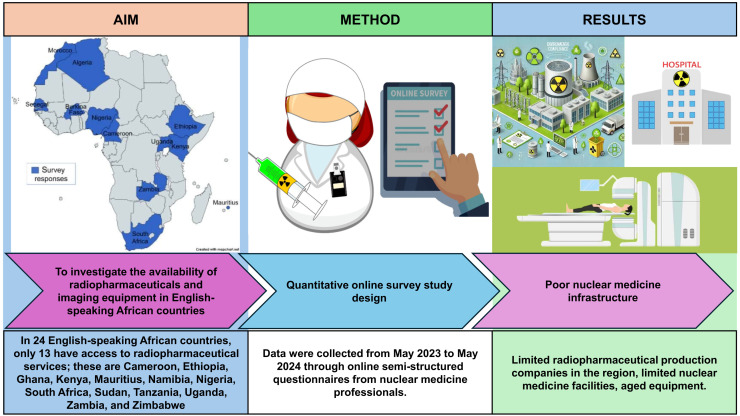

Africa’s disease profile is shifting from communicable to noncommunicable diseases, with noncommunicable diseases, such as cardiovascular diseases, neurodegenerative diseases, type 2 diabetes, and cancer, being the leading causes of morbidity and mortality on the continent (1). The growing number of older people on the continent exacerbates this health calamity, as aging is a strong determinant of cancer incidence and mortality. Though cancer may be detected at any age, many cancers are diagnosed in older people (2). Cancer is the fifth most common cause of death in Africa, with approximately 1.1 million new cases and over 700,000 deaths reported in 2020 (1).

Nuclear medicine (NM) services are essential for the management of many age-related diseases, offering advanced diagnostic modalities and treatment and palliation of malignancies (3).

In 2019, 29 African countries had at least 1 NM center, 1 had a radioimmunoassay unit, and 14 were conducting studies to determine the practicality of establishing their first NM center (4). NUMDAB, the International Atomic Energy Agency’s NM database, recorded only 74 NM centers in Africa from both the private and public sectors, 132 γ-cameras, 87 SPECT scanners, 28 SPECT/CT scanners, and 11 PET/CT scanners (5).

Africa has 54 countries (6), 24 of which are English-speaking. These include Botswana, Cameroon, Ethiopia, Gambia, Ghana, Kenya, Lesotho, Liberia, Malawi, Mauritius, Namibia, Nigeria, Rwanda, Seychelles, Sierra Leone, Somalia, South Sudan, South Africa, Sudan, Eswatini, Tanzania, Uganda, Zambia, and Zimbabwe (7,8).

The geographic distribution and availability of NM services in Africa have not yet been determined. Egypt, Morocco, and South Africa are the only African countries with research reactors. Egypt has 2 research reactors: Experimental Training Research Reactor-1 and -2, situated at the Nuclear Research Center in Inshas. Morocco has a research reactor at the National Energy Center for Nuclear Science and Technology. South Africa, the only English-speaking African country among the 3 countries, has the South African Fundamental Atomic Research Installation-1 reactor, based at the Nuclear Energy Corp. of South Africa, outside Pretoria, and it is one of the biggest producers of ^99^Mo in the world. South Africa manufactures and commercializes radiopharmaceuticals, producing radionuclides primarily through the South African Fundamental Atomic Research Installation-1 reactor and particle accelerators. Particle accelerators are widely available across the country. iThembaLabs, in the Western Cape, houses one of the largest particle accelerators in South Africa (9). Recently, the country launched Nuclear Medicine Research Infrastructure in Pretoria, a center for advancing drug discovery and development, collaborative research, and clinical trials. The facility has 2 particle accelerators and a Good Manufacturing Practices radiopharmacy (10). Kenya focuses on the production of PET radionuclides for local use. Two facilities offer NM services in Kenya, and both have particle accelerators for producing PET radionuclides for in-house consumption. Nonetheless, these facilities still have to import radiopharmaceuticals for SPECT imaging (11). Many African countries rely on the importation of radiopharmaceuticals from European countries and South Africa because of the lack of local production capacity, lack of qualified personnel, inadequate equipment, and substandard infrastructure (12,13). Much of the time, the delivery of radiopharmaceuticals to these countries is restricted because of unreliable suppliers and flight restrictions to the region (4,14).

This article reports on part of a larger study aimed to investigate access to radiopharmaceuticals in English-speaking African countries. It discusses the distribution of radiopharmaceutical services and imaging equipment among and within African countries, which will assist donors and policymakers in prioritizing resources.

## MATERIALS AND METHODS

This study was approved by Sefako Makgatho Health Science University Research Ethics Committee (SMUREC/P/92/2023:PG). Written informed consent was obtained from all participants involved in the study. This cross-sectional study collected data between May 2023 and May 2024 via an online survey with semistructured questionnaires (SurveyMonkey).

The questionnaire was divided into different sections for ease of navigation and collected information on the following: name of reporting facility and country; quantity of radiopharmaceutical equipment available at the facility and year of installation; radiopharmaceuticals for SPECT, PET, and therapy used at the facility and the country of procurement; and radiopharmaceutical cold kits used or produced at the facility.

The questionnaire was piloted among radiopharmacists and radiopharmacy students at a medical university in South Africa.

The targeted populations for this study were representatives from the African Regional Cooperative Agreement for Research, Development, and Training Related to Nuclear Science and Technology (known as AFRA) and NM facility representatives (radiopharmacists, NM technologists, medical physicists, and NM physicians from public and private sectors) in English-speaking African countries that offer NM services. Because of language barriers, only representatives from English-speaking African countries were included in the survey.

The responses were entered into an Excel (Microsoft Corp.) spreadsheet, and a descriptive analysis was performed. Identical responses from the same facilities were removed. When discrepancies in responses regarding the same facility were noted, the facility was contacted for verification.

## RESULTS

Participants’ comprehensive demographic data were not collected. Thirteen countries participated in the study (Table 1). Of the 135 NM facilities in these countries, representatives from 54 NM facilities participated in the study, yielding a 40% response rate. South Africa has 92 private NM facilities, and most did not participate in the study despite numerous invitations and telephone calls to assure them of anonymity. Thus, participation from the South African private sector was very low.

**TABLE 1.** NM Sites in English-Speaking African Countries

Country	Population*	No. active NM sites	NM sites participating in study
Cameroon	29,123,744	1	1 (100)
Ethiopia	132,059,767	2	2 (100)
Ghana	34,127,414	1	1 (100)
Kenya	56,432,944	2	2 (100)
Namibia	3,030,131	4	4 (100)
Nigeria	232,679,478	4	3 (75)
Mauritius	1,271,169	1	1 (100)
South Africa	64,007,187	109	30 (28)
Sudan	50,448,963	6	5 (83)
Tanzania	68,560,157	2	2 (100)
Uganda	50,015,092	1	1 (100)
Zambia	21,314,956	1	1 (100)
Zimbabwe	16,634,373	1	1 (100)

* Data are from (6).

Data are number or number with percentage in parentheses.

The total population of the 11 English-speaking African countries without NM services is 331,758,822. Those countries are Botswana, Gambia, Liberia, Lesotho, Malawi, Rwanda, Seychelles, Sierra Leone, Somalia, Eswatini, and South Sudan.

### Radiopharmaceutical Imaging Equipment

The availability of NM imaging equipment in a country is a major determinant of the accessibility and effectiveness of radiopharmaceutical services for patients. In English-speaking African countries, there is limited availability of imaging equipment, and, where available, it is often outdated and prone to breaking down. Table 2 details the imaging equipment available in English-speaking African countries and their age or year of installation.

**TABLE 2.** Radiopharmaceutical Equipment Type and Age as of 2023

Country	γ-Camera	Age	SPECT	Age	SPECT/CT	Age	PET	Age	PET/CT	Age
Cameroon			1	19						
Ethiopia			1	13	3	1				
Ghana			1	3						
Kenya					2	2–3			3	2–4
Mauritius			3	10–15	3	6–10				
Namibia	1	17	4	N/A						
Nigeria	1	17	4	3–15						
South Africa	8	6–13	32	1–27	25	2–17	3	N/A	15	1–16
Sudan	2	17–24	4	17–24	1	2				
Tanzania			2	10–12	1	2				
Uganda			2	5	1	1				
Zambia	1	>33								
Zimbabwe			1	9						

N/A = not available.

Age expressed in y.

Cameroon, Ghana, Zambia, and Zimbabwe have a single γ-camera per country, most of which use old technology, such as a planar 2-dimensional γ-camera, and do not yield the best diagnostic images in terms of image resolution. In the region, there are 13 planar γ-cameras (age, 6–33 y), 55 SPECT cameras (age, 3–27 y), 3 PET scanners, and 18 PET/CT scanners (age, 1–16 y). South Africa has the greatest number of cameras (83 in total), including 18 PET scanners. Kenya is the only country in the region equipped with the latest imaging technologies, such as SPECT/CT and PET/CT scanners.

### SPECT Radiopharmaceuticals

All English-speaking African countries use ^99^Mo/^99m^Tc generators for their SPECT studies. Seventeen facilities from 12 of these countries indicated using only ^99m^Tc-based radiopharmaceuticals for their SPECT studies. South Africa is the only country in the region that uses a wider variety of SPECT radionuclides in addition to ^99m^Tc, such as ^111^In, ^67^Ga, and ^133^Xe. Table 3 lists the countries that have ^99^Mo/^99m^Tc generators and the countries from which the generators are procured.

**TABLE 3.** English-Speaking African Countries Using ^99^Mo/^99m^Tc Generators and Countries of Procurement (*n* = 40)

	Country of procurement				
Country	France	Germany	Poland	South Africa	Turkey
Cameroon	1			1	
Ethiopia	1		1		
Kenya	2				
Ghana	1				1
Mauritius	1				
Namibia	1		1	2	
Nigeria				2	1
South Africa				20	
Sudan	2				
Tanzania				1	
Uganda				1	
Zambia				1	
Zimbabwe				1	

South Africa has the highest consumption of ^99^Mo/^99m^Tc generators, as it is the English-speaking African country with the largest number of NM sites. Approximately 62% of English-speaking African countries relied on a sole supplier for their ^99^Mo/^99m^Tc generators, 23% (Ethiopia, Kenya, Mauritius, and Sudan) procured their generators solely from Europe, and 38% (South Africa, Tanzania, Uganda, Zambia, and Zimbabwe) procured theirs solely from South Africa. This situation indicates the need for more local manufacturing facilities, as African countries prefer local procurement. Cameroon, Ethiopia, Ghana, Namibia, and Nigeria procured generators from multiple countries. South Africa is the only English-speaking African country that commercializes ^99^Mo/^99m^Tc generators and supplies about 62% of the countries in this region, including some of those procuring from multiple countries.

Radiopharmaceutical cold kits are components of radiopharmaceuticals that give specificity to the radionuclides. Different radiopharmaceutical cold kits are used to target different organs in the body. Table 4 lists the different radiopharmaceutical cold kits used in different countries. However, prostate-specific membrane antigen (PSMA) can also be labeled with ^18^F and ^68^Ga for PET imaging. South Africa is the only country among the English-speaking African countries that uses all the radiopharmaceutical cold kits listed in Table 4. The only 2 radiopharmaceutical cold kits used by all 13 English-speaking Africans are methoxyisobutylisonitrile and methylene diphosphonate. Methylene diphosphonate is suited for skeletal imaging, because most cancers metastasize to the skeleton, making this agent vital for bone imaging (15). Methoxyisobutylisonitrile is ideal for cardiac imaging. Because cardiovascular diseases are among the leading causes of chronic disease mortality in Africa, the use of this agent was reported for all NM sites (16,17). Ninety-two percent of English-speaking African countries use both dimercaptosuccinic acid and diethylenetriaminepentaacetic acid to examine renal structure and function.

**TABLE 4.** Types of Radiopharmaceutical Cold Kits Used in English-Speaking African Countries (*n* = 54)

Kit	Cameroon	Ethiopia	Ghana	Kenya	Mauritius	Namibia	Nigeria	South Africa	Sudan	Tanzania	Uganda	Zambia	Zimbabwe
DISIDA			1	1		4	2	18	1	1	1		
DSMA	1	2	1	2	1	4	3	17	5		1	1	1
DTPA	1	2	1	2	1	4	3	19	5		1	1	1
HMPAO					1	4	1	13			1	1	1
LeukoScan (Immunomedics)						4		8					
MAA				1	1	4	2	20	3	1		1	1
MAG3					1	4	2	23	1	1	1	1	1
Mebrofenin						2		8		2	1		1
MDP	1	2	1	2	1	4	3	28	5	2	1	1	1
MIBI	1	1	1	2	1	4	2	16	4	2	1	1	1
Nanocolloid	1			2	1	4	2	16	4	2	1	1	1
PSMA			1	1	1		1	13					
Pyp				1	1	3	2	15	1	1			
RBC					1	4	1	26		2	1		
Technegas								8					1
Tektrotyd (ROTOP Pharmaka)						4		9		1			1
Tin Colloid				1		4	1	18		2	1		
Vasculocis (Curium)								4		1	1		

DISIDA = *N*-(2,6-diisopropyl-phenylcarbamoylmethyl)iminodiacetic acid; DSMA = dimercaptosuccinic acid; DTPA = diethylenetriaminepentaacetic acid; HMPAO = hexamethylpropyleneamine oxime; MAA = macroaggregated albumin; MAG3 = mercaptoacetyltriglycine; MDP = methylene diphosphonate; MIBI = methoxyisobutylisonitrile; Pyp = pyrophosphate; RBC = red blood cell.

Few African countries have the capacity to manufacture their radiopharmaceutical cold kits, so they depend largely on importation. Table 5 lists the countries from which English-speaking African countries procure their radiopharmaceutical cold kits.

**TABLE 5.** Countries Supplying Radiopharmaceutical Cold Kits to English-Speaking African Countries (*n* = 43)

	Country of procurement					
Country	Belgium	France	Hungary	Poland	Turkey	South Africa
Cameroon		1				
Ethiopia		1			1	
Ghana					1	
Kenya	1	2			1	
Mauritius		1	1			1
Namibia			1	1		3
Nigeria					1	2
South Africa		1	2			22
Sudan				3	1	
Tanzania						1
Zambia						1
Zimbabwe						1

Fifty-four percent of English-speaking African countries source their radiopharmaceutical cold kits from more than one country (Belgium, France, Germany, Hungary, Poland, Turkey, and South Africa). South Africa is the only African country that sells radiopharmaceutical cold kits in this region, supplying 6 countries other than itself (Mauritius, Namibia, Nigeria, Tanzania, Zambia, and Zimbabwe; Table 5).

### PET Radiopharmaceuticals

PET services are not widely available in English-speaking African countries. Of the 13 English-speaking African countries, only 3 (Kenya, South Africa, and Sudan) reported providing PET services (Table 6), leaving 77% of the countries without access to PET technology.

**TABLE 6.** NM Sites with PET Scanners and PET Radiopharmaceuticals Used in English-Speaking African Countries (*n* = 17)

Country	[^18^F]FDG	[^18^F]DOPA	[^18^F]PSMA- 1007	[^18^F]DOPA	[^68^Ga]Ga- PSMA-11	[^68^Ga]Ga- DOTATATE	[^68^Ga]Ga- DOTANOC
Kenya	2		2				
South Africa	3	3	1	4	15	13	3
Sudan	N/A	N/A	N/A	N/A	N/A	N/A	N/A

The 2 NM facilities in Kenya with PET scanners listed [^18^F]FDG and [^18^F]PSMA-1007 as the only 2 PET radiopharmaceuticals currently used, and both facilities have cyclotrons; therefore, they produce their own PET radiopharmaceuticals. Sudan did not specify which PET radiopharmaceuticals it uses. South Africa is the only country that uses both ^18^F- and ^68^Ga-labeled radiopharmaceuticals. South Africa is also the only English-speaking country that commercializes the long-lived ^68^Ge/^68^Ga generators. Both Kenya and South Africa procure their PET radiopharmaceuticals locally.

### Radiopharmaceutical Therapies

For many years, radionuclide therapy has consisted primarily of radioactive iodine for thyroid cancer and thyrotoxicosis; however, in recent years, advances have been made in the discovery of new radiopharmaceutical therapies. Table 7 lists the radiopharmaceutical therapies currently used in the region.

**TABLE 7.** Radiopharmaceutical Therapies Used in English-Speaking African Countries (*n* = 34)

Country	[^225^Ac]Ac- PSMA	[^131^I]I- MIBG	[^123^I]I-Na	[^177^Lu]Lu-DOTATATE/ DOTANOC	[^177^Lu]Lu- PSMA	[^186^Re]Re sulfide	[^90^Y]Y citrate	[^90^Y]Y colloid	[^90^Y]Y microsphere
Cameroon		1	1						
Ethiopia	1		2				1		
Ghana			1				1		
Kenya		2	2	1			1		
Mauritius			1	1					
Namibia			4						
Nigeria			3			1		1	
South Africa		7	13	13	12	11	1	1	1
Sudan		3	3					3	
Tanzania		1	1						
Uganda			1						
Zambia			1						
Zimbabwe			1						

Radionuclide therapy has steadily gained popularity across English-speaking African countries, with growing use of [^177^Lu]Lu-DOTATATE, [^177^Lu]Lu-PSMA-1007, and, to a lesser extent, [^225^Ac]Ac-PSMA-167 and [^90^Y]Y microsphere. All 13 English-speaking African countries provide some form of therapy for cancer, but the use of radioactive iodine dominates as the most common radionuclide therapy. South Africa continues to be the region’s leader in the distribution and use of radiopharmaceutical therapies. Although no facility in South Africa indicated using [^225^Ac]Ac-PSMA-617, the literature has shown that this radiopharmaceutical is used and extensively researched in South Africa (18).

There was generally a poor response to where facilities procured their radiopharmaceutical therapies, with 14 indicating that they obtain [^131^I]I-Na and ^177^Lu-labeled radiopharmaceuticals from South Africa.

## DISCUSSION

The findings of this study revealed major challenges regarding radiopharmaceutical access in the 13 English-speaking African countries. Only 54% of these countries have access to radiopharmaceutical services. Even the countries with these services do not have enough NM sites to ensure that all patients can access the service. Nigeria, the most populated country, has only 4 NM sites (Table 1), giving an access ratio of 58,169,870 inhabitants per NM site. Although data were limited data from South Africa, the country with the most NM sites (*n* = 109), we found that all NM sites use a ^99^Mo/^99m^Tc generator (Table 2). Since its discovery for medical use in the 1960s, ^99m^Tc remains the mainstay radionuclide for diagnostic imaging because of its ease of in-house radiolabeling with lyophilized radiopharmaceutical cold kits for instant use, multiple oxidation states, and flexible chemistry (19,20).

South Africa, the country with the largest NM infrastructure in the region, has a SPECT/SPECT/CT camera availability of only 1camera per capita (83 cameras − 18 PET scanners = 65 cameras/64,007,187 × 1,000,000 = 1). This figure is significantly lower compared than that for Canada, which boasted an availability of 8.3 cameras per capita in 2022–2023 (21).

This study also revealed that 62% of English-speaking African countries depend on sole suppliers of ^99^Mo/^99m^Tc generators. The recent global shortage of ^99^Mo was due to the simultaneous maintenance of 3 of the global ^99^Mo reactors, coupled with another reactor experiencing mechanical failure. Additionally, the coronavirus disease 2019 pandemic, which disrupted the supply chain of these generators due to flight restrictions in many regions and made access to these generators even more challenging, exacerbated the situation (14). Five countries in this region procure ^99^Mo/^99m^Tc generators exclusively from South African suppliers, while 6 countries in the region source their generators from outside the continent.

For radiopharmaceutical cold kits, 62% of African countries use a multicountry procurement approach, and 31% depend on a sole supplier (Table 4). All countries use methylene diphosphonate and methoxy-isobutyl-isonitrile for radiolabeling with ^99m^Tc for SPECT imaging.

Noninvasive molecular imaging modalities can provide medical practitioners with a precise diagnosis before there is a physical manifestation of the disease (22). PET scanners offer greater sensitivity, which enhances early detection of disease. However, access to this service is limited in English-speaking African countries, where only 23% of the countries indicated having access to the service. This study highlights the need for policymakers to prioritize improving access to radiopharmaceutical services by scaling up and increasing the capacity of NM sites in individual countries as well as ensuring a reliable supply chain. They must prioritize and invest in the local production of PET radiopharmaceuticals. One way this can be achieved is by collaborating with or opening their borders to private investors. A successful example of this is the collaboration between a European company considered the world leader in particle-accelerator technology, Ion Beam Application, and countries like Ghana, Tanzania, and South Africa. Together, they are working to install more cyclotrons in these countries to increase access to radiopharmaceuticals (23).

A study performed by Grigoryan et al. (4) indicated that most African countries are dependent on European countries and Turkey for the procurement of radiopharmaceuticals. That was not the case in this study, as 54% of the English-speaking African countries procured their ^99^Mo/^99m^Tc generators from South Africa, while the rest procured theirs from European countries or Turkey (Table 2). However, this study investigated only 1 region of the continent, and the findings do not represent the procurement behavior of the whole continent.

The age-related mortality rate from chronic diseases is higher in sub-Saharan Africa than anywhere else in the world (17). This necessitates the intensification of early-disease detection strategies and cancer therapy programs, such as peptide receptor radionuclide therapy, by policymakers.

This study found that all English-speaking countries performed some form of cancer nuclear therapy, with most countries using older radionuclides for targeted therapy, such as ^131^I for thyroid cancer, [^90^Y]Y microspheres for hepatocellular carcinoma, and [^90^Y]Y colloids for radiosynovectomy.

This study also revealed that theranostics is an application primarily used in South Africa using [^68^Ga]Ga-DOTATATE paired with [^177^Lu]Lu-DOTATATE for neuroendocrine tumors and [^68^Ga]Ga-PSMA paired with [^177^Lu]Lu-PSMA or [^225^Ac]AC-PSMA for prostate cancer (24). Other countries, such as Ethiopia, Mauritius, and Kenya, have introduced theranostics as a treatment approach in their practices (Table 6).

## CONCLUSION

Specialized health care is pivotal in addressing the current disease burden facing Africa. Radiopharmaceutical services are crucial in the early detection of diseases, treatment monitoring, as and the noninvasive treatment of cancer. Two countries in the English-speaking African region have demonstrated promising developments in this area (Kenya and South Africa); the other countries are still lagging. The status of radiopharmaceutical services in countries like Zambia is alarming: there is only 1 NM site in the country, equipped with a single planar γ-camera that is over 33 y old. This situation signals a need for urgent intervention by policymakers or donors before the service becomes obsolete in the country. The study also revealed significant inequalities in radiopharmaceutical access among English-speaking African countries. Approximately 46% of the countries in this region have only one NM site per country. South Africa is the only country with 109 NM sites, most of which are private sector. Sole-supplier dependency is still a challenge in English-speaking African countries. Access to PET technology is very limited, with 77% of these countries—538,801,189 people—having no access to these services.

A major limitation of the study was its limited focus on a small region of the continent, making it difficult to generalize the findings to the rest of Africa.

## DISCLOSURE

This research was funded by NRF grant number NGAP23030180554 and DHET through the new Generation of Academics Program phase 7. No other potential conflict of interest relevant to this article was reported.

## Notes

KEY POINTS **QUESTION:** What is the level of radiopharmaceutical and imaging equipment availability in English-speaking African countries and from where are the radiopharmaceuticals procured? **PERTINENT FINDINGS:** An online quantitative survey study of representatives from 13 English-speaking African countries revealed that access to radiopharmaceuticals in this region is a huge challenge, as these countries are dependent on the importation of these drugs and lack imaging equipment. **IMPLICATIONS FOR PATIENT CARE:** Patients in this region will likely undergo expensive and invasive disease management and diagnosis procedures because of the lack of NM equipment and therapies.
